# Prosthetic replacement in femoral neck fracture in the elderly

**DOI:** 10.4103/0019-5413.43402

**Published:** 2008

**Authors:** Ashish Anand

**Affiliations:** *Department of Orthopedics, Primus Ortho and Spine Hospital, Chandragupta marg, New Delhi - 21, India.*

Sir,

I read with great interest the article by Marya *et al.*^1^. I must complement the authors for a well-written article. However, I need a clarification as the authors have suggested in Materials and Methods that one of the deciding factors for choosing the type of implant (cementless vs cemented) was bone stock. They have suggested that patients with better bone stock were offered cemented or hybrid hips, which is quite contrary to the classical teaching. The universal recommendation for cementless vs cemented stems is based on Dorr's classification, which is the universal standard in the West.^2^ According to this classification, Type A or Type B femurs (better bone stock) are the candidates for cementless stems, and Type C femurs are the candidates for cemented stems.^3^ The other factors that decide the type of implant are age, degree of activity, and body weight. In general, the younger, active, and heavier patients need cementless stems.^4^
